# Tropical diabetic hand syndrome

**DOI:** 10.4103/0973-3930.45273

**Published:** 2008

**Authors:** Sangeeta Tiwari, Ashutosh Chauhan, N. T. Sethi

**Affiliations:** Department of Surgery, Military Hospital, Ambala, India; 1 Department of Oncosurgery, Army Hospital (RandR), Delhi, India

**Keywords:** Diabetes, gangrene, hand, infection

## Abstract

Tropical diabetic hand syndrome (TDHS) is a terminology used to describe a specific complication affecting patients with diabetes mellitus in the tropics. The syndrome encompasses a localized cellulitis with variable swelling and ulceration of the hands to progressive, fulminant hand sepsis, potentially fatal. Since this syndrome is less recognized it is often under-reported. Authors present two cases of TDHS and emphasize on aggressive glycemic control and surgical therapy to prevent potential crippling or fatal complications.

## Introduction

“Tropical diabetic hand syndrome (TDHS) is a terminology used to describe a specific acute symptom complex found in diabetic patients in tropics which usually follows minor trauma to the hand, and is associated with a progressive synergistic form of gangrene.[1] It is both poorly understood by patients and clinicians and severe in consequence without prompt and aggressive treatment. Previous small series or case reports indicate the severe consequences of TDHS, including permanent disability and death.[2,3] We describe our experience of treating two cases of TDHS, their presentation, management and final outcomes.

## Case Reports

### Case 1:

**Figure 1:**
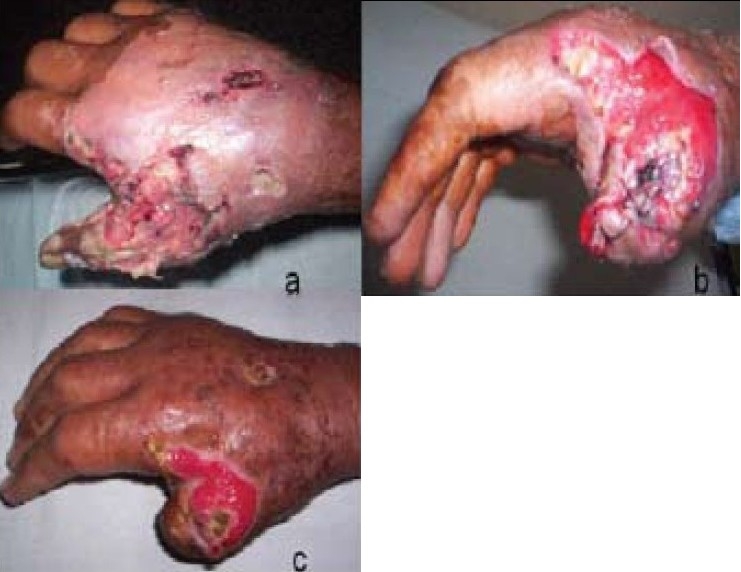
Patient presented with cellulitis and abscess of Rt hand (a) At presentation (b) Day 3: Amputation of thumb (c) Day 15: At discharge

A 45-year-old man, a known diabetic of 8-year duration, on regular medication and follow-up, sustained a superficial laceration on dorsum of (Rt) hand. He rapidly developed features suggestive of cellulitis for which he was put on some medication by a local healer. On presentation to us, he found to have diffuse cellulitis and 1st web space abscess of affected hand [Figure 1(a)]. He was put on empirical Inj. Cloxacillin, Inj. Gentamicin, Inj. Flagyl and Insulin infusion with close glucose monitoring to maintain blood sugar in range of 150–200mg %. He underwent incision and drainage of abscess, wound debridement and amputation of a nonviable 1st digit subsequently [Figure 1(a, b, c)]. Pus swab culture and culture of debrided tissue revealed Methicillin-resistant *Staphylococcus aureus* strain (MRSA) sensitive to imipenem and piperacillin only. It was allowed heal by secondary intention. At time of discharge, he had healthy granulation tissue, [Figure 1(c)].

### Case 2:

**Figure 2:**
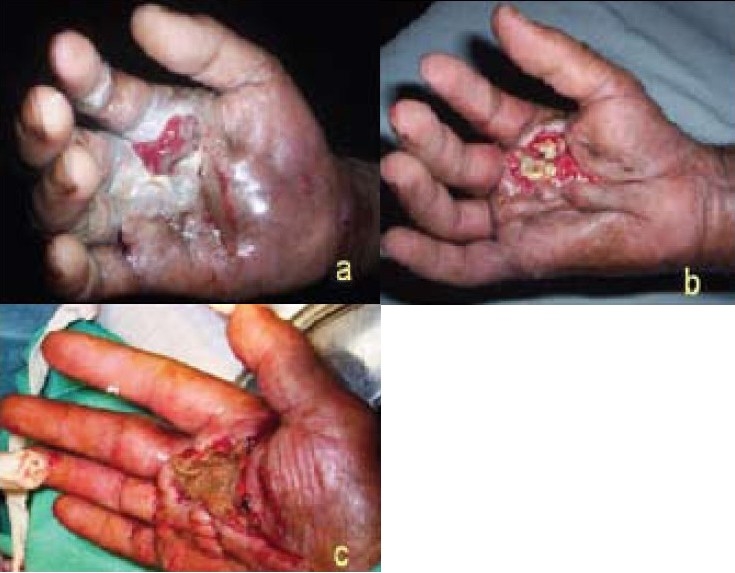
Patient presented with palmar abscess and ulceration of Rt hand (a) At presentation (b) Day 11 (c) Day 18: SSG

A 62-year-old man, a known diabetic of 10-years duration and on regular medication presented with rapidly increasing ulceration and palmar abscess of (Rt) hand [Figure 2a]. Patient did not recall any specific history of trauma. Patient had been prescribed two courses of oral antibiotics by a local practitioner before he was referred to us. He was initially put on empirical Inj. Cloxacillin Inj. Gentamicin, Inj. Flagyl and Insulin infusion. He underwent aggressive debridement and desloughing on multiple sittings over the next 15 days. The deep palmar spaces were incised, exposed and allowed to drain freely. Pus swab culture and culture of debrided tissue revealed Pseudomonas sensitive only to ticaracillin, vancomycin and augmentin. The wounds were allowed to heal by secondary intention [Figure 2(b)] and then finally underwent split skin graft cover [Figure 2(c)]. Patient thereafter underwent physiotherapy of the hand and recovered full range of movement over the next 3 months.

## Discussion

TDHS is less well recognized than foot infections and encompasses a localized cellulitis with variable swelling and ulceration of the hands to progressive, fulminant hand sepsis. It can rapidly progress to synergistic gangrene (Meleney's gangrene), affecting the entire limb and confining to the superficial fascia that can result in death within days of onset of symptoms.[1] Majority of cases have been reported from African continent[2–4] and also from India.[5] It is imperative to distinguish TDHS from the traditionally described diabetic hand syndrome, which consists of joint limitations (inability to fully extend a finger) and thickened skin of the hand, especially involving the dorsum of the fingers.[6] While peripheral vascular disease and peripheral neuropathy are well-known risk factors for diabetic foot ulcers and foot infections, neither appear to play a substantial role in the pathogenesis of TDHS.[7] There is often a history of antecedent minor hand trauma (e.g., scratches or insect bites) and as seen in our cases, presentation to hospital is often delayed due to the patients’ unawareness of the potential risks, lack of concern because the initiating trauma might have been trivial, or decision to seek initial help from local healers. Appropriate antimicrobial therapy has to be instituted immediately based on antibiotic culture and sensitivity testing. In our center, we rely on culture of tissue biopsy specimens because it has been shown that culture of tissue biopsy specimens yields a single bacterial species in >75% of cases, whereas swab cultures yield polymicrobial flora in the majority of cases, probably because of contamination.[7] Appropriate treatment for the majority of patients includes incision and drainage of the wound, debridement, or amputation. Without prompt, aggressive treatment, TDHS can lead to permanent disability, limb amputation (13% of TDHS patients require major upper limb amputation) or death.[8] The authors have learned that the surgical incision must extend along the entire area of erythema and induration because the infection often is more extensive than suspected both before and during the initial surgery. Observation, local wound care, and the administration of oral antibiotic agents are not acceptable substitutes for surgical decompression in the diabetic patient with a hand infection. These cases emphasize the need TDHS be recognized by clinicians in developing countries and treat it aggressively to prevent complications which have potential for socioeconomic burden.
